# Update on Monoterpenes as Antimicrobial Agents: A Particular Focus on p-Cymene

**DOI:** 10.3390/ma10080947

**Published:** 2017-08-15

**Authors:** Anna Marchese, Carla Renata Arciola, Ramona Barbieri, Ana Sanches Silva, Seyed Fazel Nabavi, Arold Jorel Tsetegho Sokeng, Morteza Izadi, Nematollah Jonaidi Jafari, Ipek Suntar, Maria Daglia, Seyed Mohammad Nabavi

**Affiliations:** 1 Sezione di Microbiologia DISC-IRCCS San Martino-IST University of Genoa, 16132 Genoa, Italy; anna.marchese@unige.it (A.M.); ramona.barbieri@unige.it (R.B.); 2 Research Unit on Implant Infections, Rizzoli Orthopaedic Institute, via di Barbiano 1/10, 40136 Bologna, Italy; carlarenata.arciola@ior.it; 3 Department of Experimental, Diagnostic and Specialty Medicine (DIMES), University of Bologna, Via San Giacomo 14, 40126 Bologna, Italy; 4 National Institute for Agricultural and Veterinary Research (INIAV), I.P., Vairão, 4480 Vila do Conde, Portugal; anateress@gmail.com; 5 Center for Study in Animal Science (CECA), ICETA, University of Oporto, 4051-401 Oporto, Portugal; 6 Applied Biotechnology Research Center, Baqiyatallah University of Medical Sciences, Tehran 19395-5487, Iran; nabavisf@gmail.com; 7 Department of Drug Sciences, Medicinal Chemistry and Pharmaceutical Technology Section, University of Pavia, 27100 Pavia, Italy; aroldjorel.tseteghosokeng@universitadipavia.it; 8 Health Research Center, Baqiyatallah University of Medical Sciences, Tehran 19395-5487, Iran; morteza_izadi@yahoo.com (M.I.); mjafari@hs.uci.edu (N.J.J.); 9 Department of Pharmacognosy, Faculty of Pharmacy, Gazi University, Etiler, Ankara 06330, Turkey; kriptogam@gmail.com

**Keywords:** antibacterial, antimicrobial, cymenes, monoterpenes

## Abstract

p-Cymene [1-methyl-4-(1-methylethyl)-benzene] is a monoterpene found in over 100 plant species used for medicine and food purposes. It shows a range of biological activity including antioxidant, anti-inflammatory, antinociceptive, anxiolytic, anticancer and antimicrobial effects. This last property has been widely investigated due to the urgent need for new substances with antimicrobial properties, to be used to treat communicable diseases whose diffusion in developed countries has been facilitated by globalization and the evolution of antimicrobial resistance. This review summarizes available scientific data, as reported by the most recent studies describing the antimicrobial activity of p-cymene either alone, or as the main component of plant extracts, as well as addressing the mechanisms of action of cymenes as antimicrobial agents. While p-cymene is one of the major constituents of extracts and essential oils used in traditional medicines as antimicrobial agents, but considering the limited data on its in vivo efficacy and safety, further studies are required to reach a definitive recommendation on the use and beneficial effects of p-cymene in human healthcare and in biomedical applications as a promising candidate to functionalize biomaterials and nanomaterials.

## 1. Introduction

Plant extracts and their secondary metabolites are rich sources of antimicrobial substances, including coumarins and psoralens, acetylenes, flavonoid and non-flavonoid polyphenols, and terpenes [1,2,3,4,5]. Monoterpenes (i.e., eucalyptol, borneol, camphor, bornylacetate, carvacrol, (−)-menthol, γ-terpinene, (+)-α-pinene, (−)-β-pinene, and p-cymene) are the most important constituents of essential oils produced through liquid extraction and steam distillation of edible and medicinal plants [1]. p-Cymene [1-methyl-4-(1-methylethyl)-benzene] is an alkyl-substituted aromatic hydrocarbon found in nature, whose benzene ring features the substitution of a methyl and an isopropyl group, and which is considered to be the most important monoterpene compound occurring in aromatic plants, such as thyme and oregano. p-Cymene is found in more than 100 plant species, including many belonging to Thymus, Origanum, *Ocimun* (Lamiaceae), *Eucalyptus* (Mirtaceae), *Protium* (Burseraceae), and *Artemisia* (Asteraceae) genus [6]. This compound shows a variety of biological activities which include antioxidant, antinociceptive, anti-inflammatory, anxiolytic, anticancer and antimicrobial activities. In fact, recent in vivo investigations performed on experimental animal model systems (adult male Swiss mice), showed that p-cymene increases the activity of antioxidant enzymes, reducing oxidative stress [7,8]. In addition, it shows anti-inflammatory activity, being able to modulate cytokine production (tumor necrosis factor-α-TNF-α, interleukin-1β-IL-1β, interleukin-6-IL-6) in vitro (murine macrophage-like cell line RW 264.7) and in vivo (Female C57BL/6) by inhibiting nuclear factor-κB (NF-κB) and mitogen-activated protein kinase (MAPK) signaling pathways involved in synthesis of pro-inflammatory cytokines [9]. The anti-inflammatory activity of p-cymene at least partly justifies its antinociceptive activity, as recorded in several in vivo studies performed on murine model systems [8,10,11]. Moreover, recent investigations report the antitumor activity of p-cymene and metal p-cymene complexes such as rutenium (II)- and osmium (II)-p-cymene complexes, which inhibit tumor proliferation through an antiangiogenic mechanism, cancer cell cytotoxicity, and anti-adhesion activity [12,13,14]. p-Cymene is the main antimicrobial compound in thyme, and a large body of evidence suggests that this monoterpene possesses antibacterial, antiviral and antifungal activities. This review reports the available data on the mechanisms of action of cymenes and summarizes major studies describing their antimicrobial activity, both alone or as main components of plant extracts, from the past five years.

## 2. Chemistry

**Figure 1:**
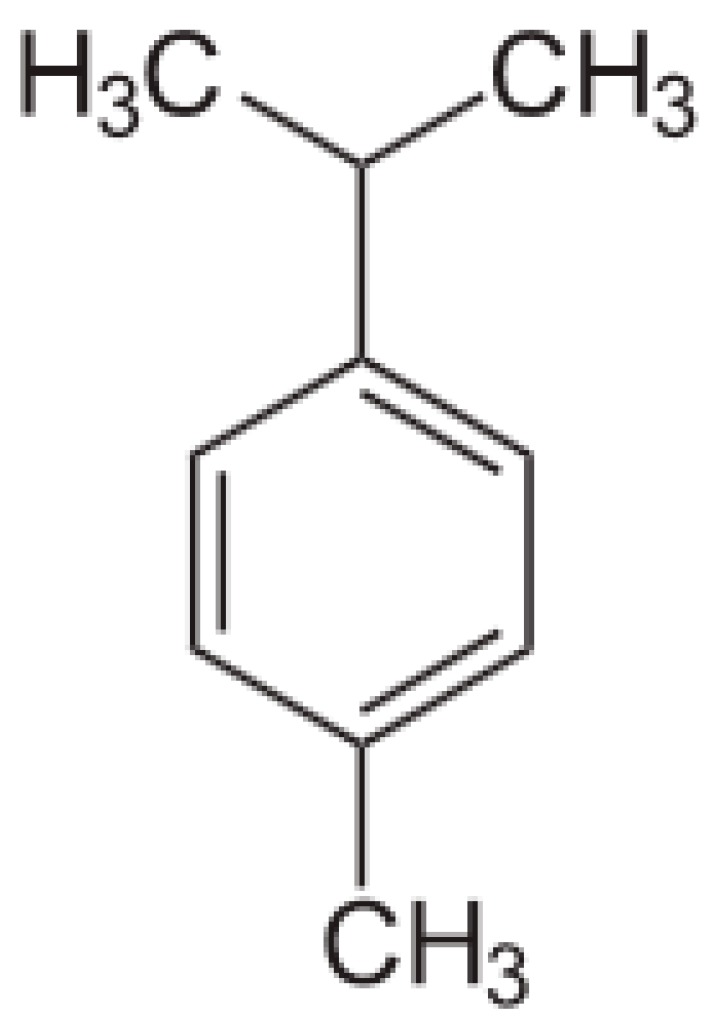
Chemical structure of p-cymene [17].

p-Cymene (also known as 4-isopropyltoluene, 1-isopropyl-4methylbenzene or 1-methyl-4-isopropylbenzene) is a naturally occurring compound, although it has two further geometric isomers, m-cymene (with meta-substituted alkyl groups) and o-cymene (with ortho-substituted alkyl groups) which do not occur naturally [5,15]. p-Cymene is related to the monoterpenes. Monoterpenes themselves belong to the “terpenes”, a larger class of organic compounds, which are the most representative components of essential oils [8,16]. Figure 1 shows the chemical structure of p-cymene, indicating the benzene ring with methyl and isopropyl substitutions. Some of the physical properties of this molecule are compiled in Table 1.

Medicinally, p-cymene is used to prevent coughs and eliminate phlegm [15] as well as being a flavouring agent, and being used in the production of fungicides and pesticides [10,18]. It is considered to be “generally recognised as safe” (GRAS) by the U.S. Food and Drug Administration [19]. Its potential in the prevention of protein glycation mediated diabetic complications has also been confirmed [15], and it has been suggested as an in vivo antioxidant compound due to its ability to reduce the formation of oxygen and nitrogen reactive species, acting as a potential neuroprotective agent in the brain. Therefore, it could be involved in the treatment of oxidative stress related diseases [7]. p-Cymene also acts as an analgesic/antinociceptive and anti-inflammatory [9,10,11] and has a vasorelaxant effect in rat mesenteric artery and aorta [20,21]. The odour of p-cymene has been described as woody and spicy [22]. Beaulieu & Stein-Chisholm (2016) [23] have compiled the attributes of p-cymene from different sources as citrus, solvent, gasoline, kerosene, fresh, woody, spice, cumin, oregano, cilantro, green pepper and rancid.

## 3. Sources

p-Cymene has been identified in the essential oils of more than 100 plants and 200 foods. Table 2 and Table 3 report the levels of p-cymene found in essential oils and plant-based foods, respectively. The composition and content of essential oils depend on several factors affecting the plant at harvest, including age, genetic factors, ripening stage, edaphoclimatic conditions and season of collection [24]. Therefore, the controlled culture of aromatic plants is recommended over the use of wild populations, in order to produce essential oils to be used in the food, pharmaceutical or cosmetic industries. Besides essential oils, p-cymene occurs in herbal drugs obtained from traditional medicinal plants. In fact, it is a component of folium eucalypti, which consists of the dried leaves of *Eucalyptus globulus* Labill (Myrtaceae) [25], fructus anethi, the dried ripe fruits of *Anethum graveolens* L. (Apiaceae) [26], and Cortex Magnoliae, the dried bark of *Magnolia officinalis* Rehder and Wilson, and *M. obovata* Thunb [27].

## 4. Bioavailability and Synthesis

One of the major limitations of p-cymene for pharmaceutical applications is its short half-life [16]. p-Cymene is rapidly absorbed into the circulation system (time of maximum concentration, T max = 0.33 ± 0.11 h) following oral administration of essential oil from *Chenopodium ambrosioides* L., and it is also eliminated rapidly in vivo, as indicated by its elimination half-life (T ½ = 0.44 ± 0.07 h) [39]. The mean plasma concentration vs time profile of p-cymene shows a double-peak which might be due to distribution, reabsorption, enterohepatic circulation, and interaction with multiple compounds present in formula [39].

In view of this short half-life, possible drug delivery systems for p-cymene have been studied [40]. Martins et al. [41] successfully encapsulated thymol and p-cymene within polylactide microparticles in order to protect these active agents and to provide a controlled release.

Due to industrial interest in p-cymene in the synthesis of pesticides, fungicides, perfumes, fragrances and as a starting material for the synthesis of p-cresol, which is in turn used in the production of antioxidants like butylated hydroxytoluene (BHT) [42], the synthesis of p-cymene has been the target of several studies. According to Kamitsou et al. (2014) [43], p-cymene is conventionally produced by the Friedel-Crafts alkylation of toluene with isopropanol, or of benzene with methyl or isopropyl halides. The catalysts of these reactions are generally solutions of HCl acid with AlCl_3_, BF_3_ or H_2_SO_4_. However, this presents several limitations: (i) they use highly toxic substances (benzene, toluene and acids); (ii) problems with corrosion and disposal due to the use of acids; (iii) the separation of reagents is difficult and time consuming, as it takes place in a liquid phase by means of a batch reactor; (iv) significant amounts of o- and m-cymene are produced. Thus, alternatives for the production of p-cymene have been investigated. Alsalme et al. [44] studied the production of p-cymene through the isomerisation of gas phase α-pinene above solid heteropoly acid catalysts at 200 °C and ambient pressure in a fixed-bed continuous flow reactor, while Al-Wadaani et al. [45] studied the production of p-cymene from α-pinene through a one-step dehydroisomerisation using Zn(II)–Cr(III) mixed oxide as a possessing acid. Another alternative could be to use α-limonene, an economic by-product of the orange juice production and paper industry. Kamitsou et al. (2014) [43] found that titanium oxide was a very good catalyst of this reaction at 300 °C and at atmospheric pressure. In a study carried out by Dávila et al. [46], the production of p-cymene and pectin from orange peel was evaluated from a process, economic, and environmental point of view and it was marked as a possible way to increase the added value of essential oils from orange peel.

## 5. Spectrum of Activity

### 5.1. Antibacterial Activity

In 2013 Hashemi et al. [47] tested essential oils and methanolic extracts of *Echinophora platyloba* D.C for in vitro antibacterial activity against certain pathogenic bacteria present in food. The major component of these essential oils and methanolic extract was o-cymene (26.51% and 28.66% respectively). *Listeria monocytogenes* and *Staphylococcus aureus* were the most affected by both essential oils (minimum inhibitory concentration (MIC): 6250 and 12,500 ppm respectively) and methanolic extract (MIC: 25,000 ppm); of the Gram-negative strains tested, *Escherichia coli* was inhibited at a MIC value of 50,000 ppm by essential oil, while methanolic extract showed no antibacterial activity. *Salmonella typhimurium* was not inhibited by any compounds. In 2014 Bukvički et al. [48] studied the in vitro activity of *Satureja horvatii* oil against some Gram-positive and Gram-negative bacteria and yeast strains. The main component of *S. horvatii* was p-cymene (33.14%) followed by thymol (26.11%). The MIC against bacteria ranged from 0.03 mg/mL for *E. coli* and *S. typhimurium* to 0.57 mg/mL for *L. monocytogenes*. Among yeasts, MIC ranged from 0.56 mg/mL (*Pichia membranaefaciens*) to 2.23 mg/mL (*Zygosacharomyce bailii* and *Aureobasidium pullulans*). Another study [49] investigated the essential oil of *Glossogyne tenuifolia* for antimicrobial activity in vitro and food systems, and its individual compounds (including p-cymene) against certain common food pathogens. p-Cymene was found to be the most common compound in this oil. A minimal bactericidal concentration equal to 12 mg/mL of p-cymene completely inhibited *E. coli* O157:H7, *Vibrio parahaemolyticus*, *L. monocytogenes*, and *S. enterica*. *S. aureus* and *Streptococcus mutans* showed a minimal microbicidal concentration of 6 mg/mL, while *S. sanguinis* was inactivated by a concentration of 3 mg/mL of p-cymene. The study also investigated the synergistic interactions of the compounds found to be most active (4-terpineol, linalool, α-terpineol and p-cymene) against the same pathogens. p-Cymene, in combination with these other molecules, showed additive effects against *S. enterica*, *S. aureus*, *S. sanguinis* and *S. mutans*, with a fractional inhibitory concentration index (FICi) of 1 for all associations, while against *E. coli* O157:H7, *L. monocytogenes* and *V. parahaemolyticus* the components showed indifferent interaction with a FIC value of 1.5. However, p-cymene was not found to be the cause of the antimicrobial activity of this essential oil.

The antibacterial activity of carvacrol and p-cymene (a precursor of carvacrol) was studied against the foodborne microorganism *V. cholerae* to evaluated the potential use of these compounds as preservative agents. Carvacrol showed a good inhibitory effect against *V. cholerae*, while p-cymene does not demonstrate this activity. However it is interesting to note that p-cymene can enhance the inhibitory effects of carvacrol when the two compounds are used together. The synergistic in vitro effect of p-cymene plus carvacrol could suggest the possible use of the combination in inhibiting *V. cholerae* and other foodborne pathogens in food [50].

Another study [51] demonstrated that *Origanum dictamnus* essential oil and its main constituents (carvacrol 52.2%, γ-terpinene 8.4%, p-cymene 6.1%, linalool 1.4% and caryophyllene 1.3%) were active against common food spoilage and pathogen strains. Against *S. enteritidis, E. coli*, *S. aureus* and *S. epidermidis*, p-cymene was the least active compound with MICs of 0.527% (v/v), 0.492% (v/v), 0.598% (v/v) and 0.608% (v/v) each, with the most active being carvacrol.

Andrade-Ochoa et al. (2015) [52] investigated the antimycobacterial effects of various essential oils including p-cymene, thymol and carvacrol against *Mycobacterium tuberculosis* and *M. bovis*. p-Cymene was found to be the terpene with lowest antimycobacterial activity (MIC of 91.66 μg/mL for both *M. bovis* and *M. tuberculosis*), while thymol and carvacrol were the most active terpenes (thymol MICs of 0.78 μg/mL for *M. tuberculosis* and 2.02 μg/mL for *M. bovis*; carvacrol MICs of 2.02 μg/mL for *M. tuberculosis* and 5.20 μg/mL for *M. bovis*). p-Cymene and the other components of *Monarda punctata* essential oil were investigated for antibacterial activity targeting *E. coli* and some common respiratory pathogens [53]. p-Cymene, thymol and limonene were the major compounds of *M. punctata* essential oil. *S. pyogenes*, methicillin-resistant *S. aureus* (MRSA) and *Haemophilus influenzae* were the most susceptible strains showing lowest MIC values, while *S. pneumoniae* and *E. coli* were the most resistant bacteria.

In the study of Patil et al. [54], the chemical composition of the essential oils from the two Indian spices *Cuminum cyminum* (cumin) and *Trachyspermum ammi* (ajowan) has been determined by gas chromatography-mass spectrometry. The major chemical components detected were cuminaldehyde and 2-caren-10-al in the cumin essential oil, while p-cymene and thymol were found in ajowan essential oil. The antibacterial activity of these essential oils was evaluated against several Gram-positive and Gram-negative bacteria. Both the oils exhibited a strong antibacterial effect against most of the tested bacteria. Furthermore, cumin and ajowan oils demonstrated remarkable antibacterial activity against *Salmonella enterica* seroval *Typhi*. Ajowan oil exhibited a wider spectrum of activities against both the Gram-positive and Gram-negative organisms when compared with cumin [54]. An antibacterial activity of oregano essential oil against planktonic *S. aureus* and *S. epidermidis*, including methicillin resistant strains has been described by Nostro et al. [55].

### 5.2. Antifungal Activity

In 2013, Aznar and colleagues [56] studied the growth of *Candida lusitaniae* under various concentrations of natural compounds including carvacrol, thymol, and cymene in order to evaluate their possible use as food preservatives. The growth rate of *C. lusitaniae* was reduced in the presence of these molecules, while the lag time increased with increasing concentrations. All molecules tested inhibited yeast growth for at least 21 days at a concentration of 1 mmol/L. The delay increased up to 45 days in the presence of cymene. Cymene was more effective than thymol and carvacrol at higher concentrations (0.2–0.5 mmol/L). In the same year, another research group [57] studied the effect of some herbal essential oils against ovine dermatophytes. *Thymus serpillum* and *Origano vulgare* essential oils showed the lowest MIC (0.1% and 0.5%), while p-cymene, that was one of the main component of these essential oils, showed no specific anti-dermathophytes activity with a MIC and a minimum fungicidal concentration (MFC) >8%.

p-Cymene does not seem to have activity against filamentous fungi; the results of a study conducted by de Lira Mota et al. (2012) [58] showed that p-cymene does not possess antifungal activity against *Rhizopus oryzae* (MICs: >1024 µg/mL for all strains). p-Cymene also showed no antifungal activity against *Aspergillus niger* (MIC: >300 µL/mL) [59].

Regarding the antifungal activity of essential oils containing high percentage of cymene, in 2014 Kedia and colleagues [60], studied the antifungal properties of *Ciminum cymininunm* L. seed essential oil against *A. flavus* strain LHP(C)-D6. The chemical characterization by gas chromatography/mass spectrometry (GC/)MS) analysis of these seed oil revealed the presence of many compounds, among them the most represented was cymene (47.08%). This essential oil showed high antifungal activity, indeed the MIC that inhibited the growth of *A. flavus* was 0.6 μg/mL, while the fungicidal concentration was 0.9 μg/mL. Given the low MIC values, the authors suggested that this essential oil could be useful for the permanent disinfection of food borne fungi from food items. Other authors [51] also evaluated the activity of *Origanum dictamnus* essential oil (containing 6.07% of p-cymene), against *Saccharomyces cerevisiae* and *A. niger*, microbes used as model system in food spoilage. The results obtained with the disk diffusion method, indicated that both fungi tested were sensitive to this essential oil.

### 5.3. Antiparasitic Activity

In 2013, a research group [60] investigated the in vitro antiparasitic activity of a series of chelating cationic ruthenium(II)-arene complexes on thiosemicarbazone scaffolds against two *Plasmodium falciparum* strains (chloroquine-sensitive and chloroquine resistant) and against a *Trichomonas vaginalis* strain. The investigation showed that complexes containing the p-cymene were more active than those containing benzene. In 2014, an investigation conducted by Kpadonou Kpoviessi (2014) [61] studied the antitrypanosomal and antiplasmodial activities in vitro of essential oils and crude extracts from *Ocimum gratissimum* against *Trypanosoma brucei brucei* and *P. falciparum*. p-Cymene represents about 31.53% of the essential oil extracted from aerial parts of *O. gratissimum* harvested in the pre-flowering stage and 28.08% of those in the full flowering stage, and showed moderate activity in this case (sample concentration provided death of 50% of parasites, with a half maximal inhibitory concentration (IC50) of 76.32 μg/mL).

### 5.4. Anti-Biofilm Activity

As an extension of the work on the efficacy of the oregano essential oil components against planktonic methicillin-resistant staphylococci [55], it has been demonstrated that these substances are able to inhibit the growth of staphylococcal preformed biofilms, and even to interfere with biofilm formation during planktonic growth [62]. The oregano oil is characterized principally by carvacrol and thymol and by their two precursors, c-terpinene and p-cymene [63]. The antimicrobial activity of oregano oil is mostly attributed to the action of its components carvacrol and thymol, which exhibited significant bactericidal activity when tested separately [64].

By scanning electron microscopy (SEM) analysis, a very high inhibitory effect by Eucalyptus essential oil on biofilm formation by *Proteus mirabilis* on urinary catheters was demonstrated. Cymene is present among the major fractions of Eucalyptus essential oil, to which the anti-biofilm properties were ascribed [65]. Besides the anti-biofilm effect on urinary catheters, Eucalyptus oil has been demonstrated to reduce biofilms formed by *S. epidermidis* on the skin [66].

Oral candidiasis is an opportunistic infection of the oral cavity, which usually occurs in the immunocompromised individuals. The effects of *Satureja hortensis* L. essential oil on the planktonic form, biofilm production, and mature biofilms of *C. albicans* from buccal lesions of HIV+ individuals have been investigated. This essential oil has thymol, gamma-terpinen, carvacrol and p-cymene among its most abundant constituents. The biofilm formation by *C. albicans* on polystyrene was drastically reduced by *Satureja hortensis* L. essential oil. At sub-MIC concentration, SEM analysis revealed loosening of cells, deformity of three dimensional structures of biofilms, and shrinkage in cell membranes of sessile cells. These observations suggest a potential exploitation of this oil as a natural anti-biofilm product in the treatment of buccal cavity lesions caused by *C. albicans* [67]. Analogously, the work of Khan et al. demonstrated an attenuation in the biofilm production by *C. albicans* (*C. albicans* 04 and *C. albicans* SC5314) in the presence of essential oils. Biofilm production was reduced maximally by thymol [68].

Very recently, it has been observed that p-cymene reduces biofilm formation in *Burkholderia xenovorans*. This effect has been ascribed to the accumulation of p-cymene in bacterial membrane and to the changes in the membrane structure induced by this aromatic compound [69].

### 5.5. Anti-Inflammatory Activity

In addition to the antibacterial and anti-biofilm activities of terpenes, in particular of p-cymene, an anti-inflammatory activity has also been proved. Anti-inflammatory compounds, both steroidal and nonsteroidal, are the major routes for the treatment of inflammatory disorders. However, the use of synthetic anti-inflammatory molecules is associated with serious common side effects, including gastric irritation, ulceration, bleeding, renal failure and hepatic damage [70,71].

In recent years, phytochemicals derived from plants have gained increased attention due to their safe toxicological profiles in respect to non-steroidal and steroidal drugs and their protective effects [70]. The recent advances in the preparation and characterization of nanosized vectors able to release phytochemicals endowed with anti-inflammatory activity and the range of new delivery technologies are presented and discussed in the cited review [72].

### 5.6. Anti-Infective and Anti-Inflammatory Biomaterials

Phytochemicals have promising potential to prevent and/or treat inflammatory and biofilm-associated diseases. Drug delivery systems based on nanomaterials can potentiate the solubility and stability of phytochemicals, improve their absorption, protect them from premature enzymatic degradation or metabolism in the body, and extend their circulation time, limiting their side effects.

A comprehensive review on nanosized delivery systems for plant drugs has been presented by Bonifácio et al. [73].

Different kind of nanomaterials have been proposed to be conjugated with monoterpenes, such as metals (particularly silver nanoparticles), polymers (mainly resorbable polymers), and chitosan (the most important derivative of chitin, a natural polymer obtained from marine crustacean shell).

The “green” synthesis of metal nanoparticles arouses great interest due to the advances in ecofriendly technologies in material science. An interesting example is the one step synthesis of antibacterial silver nano/microparticles using extract of *Trachyspermum ammi*. The extract, containing p-cymene together with thymol and γ-terpinene, was found to be a valuable agent for the formation of biocompatible silver nanoparticles. The obtained nanoparticles were triangular shaped and smaller than 100 nm [74].

Besides p-cymene, also carvacrol has been studied for its antibacterial activity when conjugated with nanomaterials. Carvacrol-chitosan nanoparticles, exhibiting a spherical shape with an average diameter of 4–80 nm, have shown antimicrobial activity against *S. aureus*, *Bacillus cereus* and *E. coli* [75].

A ionic amphiphilic chitosan derivative obtained by the interaction between chitosan and oleic acid has been recently proposed to stabilize the nanoemulsion of the essential oil *Cymbopogon citratus* (DC.) Stapf (*Lemongrass*), thus improving its delivery. The combination of spontaneous emulsification process with chitosan oleate amphiphilic properties resulted in: (i) stable dispersion of a few hundred nanometer droplets; (ii) maintenance or improvement in the essential oil antimicrobial activity towards nine bacterial and ten fungal strains; (iii) biocompatibility of the nanoemulsion (cytotoxicity test performed on four different cell lines) [76].

Phytochemical-based polymers can find applications as biomedical materials and, more importantly, they greatly contribute to the new concept of sustainable polymer chemistry. In particular, the design and preparation of terpene-based polymers involve different chemical strategies and a wide variety of polymerization techniques [77].

Carvacrol has attracted attention for its ability to promote the disruption of microbial biofilm. By encapsulation of carvacrol in poly(*dl*-lactide-*co*-glycolide (PLGA) resorbable nanocapsules a suitable drug delivery system has been obtained, representing a starting point for new therapeutic strategies against biofilm-associated infections [78].

Also, poly(lactic acid) nanofibrous resorbable membranes produced by electrospinning and solution blow spinning (SBS) have been used as carriers of an antimicrobial natural plant oil such as linalool [79], a scented terpene alcohol found in many flowers and spice plants (*Lavender*, *Coriander*, *Basil*).

A more complex system has been described, consisting in hybrid poly(lactic acid) (PLA) fibres loaded with highly crystalline cellulose nanowhiskers (filamentary crystal with cross sectional diameter ranging from 1 to 100 nm) by a novel solution blow spinning method. Carvacrol was incorporated as antimicrobial agent, demonstrating a biocide effect against *L. monocytogenes* [80]. Carvacrol has been also loaded on polyhydroxybutyrate nanoparticles exhibiting an antimicrobial activity against *E. coli* [81].

In a very recent study, clove oil, together with hydrolysed chitosan and turmeric powder, was used to formulate an antimicrobial coating for the polyethylene terephthalate (PET) and polyamide (Nylon 6) surgical sutures, with the aim of preventing wound site infections. Interestingly, the coated sutures showed antibacterial activity against *S. aureus* and even a (slight) improvement of tensile and knot strength properties. [82].

The use of antibacterial biomaterials, biomaterials surfaces, and coatings based on natural bactericidal substances produced by plants is a new fascinating approach. Over millions of years, living organisms have developed multifaceted and successful strategies to efficiently prevent the colonisation of their surfaces by pathogens. These strategies can be mimicked to create a new generation of bio-inspired antibacterial and biofilm-resistant surfaces [83].

### 5.7. Mechanism of Action

In a study conducted by Ultee et al. [84], the modes of action of cymene and carvacrol were investigated using *B. cereus*, a food borne pathogen, as a model. The study investigated the effects of natural compounds on liposomal membrane expansion, looking into their influence on membrane potential, changes in intracellular pH, influences on the amount of ATP, and effects on the growth of *B. cereus*. Carvacrol and cymene added to liposomes both caused an expansion of the cytoplasmic membrane, but the degree of expansion caused by cymene was 2.7 times greater than the expansion due to carvacrol. Both molecules induced a reduction in membrane potential, but the cymene concentrations required were higher than those of carvacrol. Cymene had no effect on the pH gradient across the membrane at concentrations from 0.5 mM to 2 mM, while at a concentration of 1 mM, carvacrol was able to eliminate the pH gradient. Moreover, it was shown that concentrations of 1 and 2.4 mM of cymene had no effect on the extra- and intra-cellular ATP levels. Conversely, carvacrol caused a decrease in intracellular ATP levels. Interestingly, it was demonstrated that the hydroxyl group present in carvacrol plays a vital part in exerting its antimicrobial effects. Carvacrol was able to inhibit *B. cereus* growth at concentrations of 0.75 mM and above, while cymene, lacking in the hydroxyl group, was unable to inhibit strain growth from 0.5 mM to 10 mM. The antimicrobial activity was affected by the presence, or lack of, the hydroxyl group rather than its position in the benzene ring.

More recently, Cristani et al. (2007) [85] evaluated the damage caused by some monoterpenes such as carvacrol and its precursor p-cymene, on biomembranes of *E. coli* and *S. aureus*. This research suggests that the antimicrobial effects of p-cymene and carvacrol could be due to a perturbation of lipids in the bacterial membrane. The ways in which carvacrol and p-cymene impact protein synthesis and cell motility in *E. coli* O157:H7 strain were also recently investigated by Burt and team [86]. The presence of sublethal concentrations of carvacrol (1 mM) overnight led to increases in heat shock protein 60 and decreases in the synthesis of flagellin, producing nonmotile bacteria, while p-cymene had an insignificant effect on the synthesis of proteins. *E. coli* O157:H7 cells in the exponential phase were subjected to a 3-h treatment of increasing concentrations of p-cymene and carvacrol, which yielded an increase in HSP 60 levels and a decrease in motility which correlated with the increased concentrations used. However, the flagella were not shed.

Li et al. (2014) [53], studied the morphological changes induced by treatment, with p-cymene and other essential oils of *Monarda punctata*, of the bacterial architecture of *S. pyogenes* and MRSA using scanning electron microscopy, also studying the generation of ROS in the treated cells. The SEM observation showed that damage induced by treatment with essential oils was dose-dependent; over 95% of bacterial cells were killed with a treatment time of 12 h at a concentration of 90 μg/mL of essential oil. The treated bacterial cells appeared swollen after 12 h, while no change in morphological structure was observed in untreated cells. Elevated levels of ROS were detected and measured after 4 h of treatment with essential oil.

## 6. Conclusions

In recent years, with the increasing incidence of antimicrobial resistance, many studies have been carried out on the antimicrobial activity of bioactive compounds. Natural compounds extracted from various plants, food and beverages, could provide possible alternatives to standard antimicrobial agents. It is well known that some commercial essential oils, especially those extracted from thyme or oregano, have antimicrobial capacities against certain food-borne bacteria. These properties are often attributed to their primary bioactive constituents.

This review summarizes the studies describing the antimicrobial activity monoterpenes, especially p-cymene, published in the past five years, either alone or as a main constituent of plant extracts. Most studies evaluated the antimicrobial activity of some essential oils and or plant extracts containing various phytochemicals, including p-cymene, but only few researches have analysed the antimicrobial activity of p-cymene alone.

Based on literature data, we can conclude that p-cymene is one of the major constituents of the extracts and essential oils obtained from many medicinal and food plants. p-Cymene uses as antimicrobial agent (especially in topical application for symptomatic treatment of common skin disorders, treatment of wounds, and vaginitis) are well described in pharmacopoeias and in traditional medicine, but are often unsupported by clinical data.

The literature data reported in this review show that when used alone, p-cymene is not the main compound conferring the antimicrobial activity of essential oils and/or plant extracts. In particular, several authors demonstrated that p-cymene is less efficient as an antimicrobial agent than its derivative carvacrol. Nevertheless, based on literature reports, we can highlight that p-cymene enhances the activity of other antimicrobial agents through synergism, antagonism and additive effects. Because of the potential cytotoxicity of several antimicrobial substances, especially found in essential oils, the finding that p-cymene improves the antimicrobial properties of other substances, such as i.e. carvacrol, 4-terpineol, and nisin [87,88,89,90] could be of great interest, especially in cosmetic and pharmaceutical fields, since p-cymene addition could reduce the concentration of other antimicrobial compounds. Regardless, the interaction among essential oil individual components and p-cymene requires further research to elucidate the mechanism underlying this biological activity. In addition, the evaluation of data from the literature highlights that p-cymene can reduce biofilm formation. This aspect needs further study, since biofilm formation is important for the persistence of pathogens under environmental conditions, inducing colonization in the host by microorganisms and increasing their resistance to antimicrobial agents.

Microbial contamination of food products is an important problem, especially in developing countries. For the use of monoterpenes, and especially p-cymene, in food products, data from the literature showed that essential oils and plant extracts, containing various phytochemicals including p-cymene, can be used as a fungicide or herbicide, as well as an insecticide. Further studies are needed to elucidate the role of p-cymene and to understand its biological activity for the purpose of developing new natural antiseptics for the food industry.

Finally, p-cymene appears a promising phytochemical to be incorporated into advanced polymers for anti-infective and anti-inflammatory biomaterials and nanomaterials.

Concluding, we advise ongoing focus on toxicological studies and clinical trials on a range of infectious diseases, aiming to evaluate the efficacy of p-cymene, both alone and as a constituent of plant extracts, and to determine a safe and efficient dose.

## Figures and Tables

**Table 1:** Some physical properties of p-cymene [17].

Physical Property	Value
Molecular Weight	134.22
Boiling Point	177.1 °C
log P (octanol-water)	4.1
Water Solubility	23.4 mg/L
Vapor Pressure	1.46 mm Hg
Henry’s Law Constant	0.011 atm-m^3^/mole at 25 °C
Atmospheric OH Rate Constant	1.51 × 10^−11^ cm^3^/molecule-sec at 22 °C

**Table 2:** p-Cymene levels in some essential oils.

Matrix	Content	Reference	Comments
Essential Oils			
*Chenopodium ambrosioides* L.	16.20%	[28]	from Madagascar
Grapefruit peel essential oil	0.12%	[29]	
*Thymus kotschyanus*	9.80%	[30]	
*Thymus vulgaris* (thyme)	15.2%	[30]	
Orange peel essential oil	0.23–9.84%	[31]	
Tangerine	4.70%	[32]	
*Thymus vulgaris* ct. Thymol (red thyme)	5–10%	[33]	from Spain
*T. vulgaris*	5–10%	[33]	from Morocco
*Oreganum vulgare* L. Subsp. Glandulosum (Desf.)	11.5–35.7%	[34]	from Nefza (Tunisia) and 3 different harvest years (2007, 2008, 2009)
*Oreganum vulgare* L. Subsp. Glandulosum (Desf.)	27.3–46.3%	[34]	from Krib (Tunisia) and 3 different harvest years (2007, 2008, 2009)
*Thymus vulgaris*	45.90%	[35]	
*Ocimum gratissimum*	14.06%	[35]	
*Mentha arvensis* L.	0.11%	[36]	
*Mentha piperita* L.	0.12%	[36]	
*Rosmarinus officinallis* L.	1.03%	[24]	
*Cinnamomum zeylanicum* Blume	1.16%	[24]	

**Table 3:** p-Cymene levels in some food matrices.

Fruits, Plants or Vegetables			
Star apple fruit (*Chrysophillum albidum*)	0.01 mg/kg	[22]	bitter and sour varieties
Star apple fruit (*Chrysophillum albidum*)	0.95 mg/kg	[22]	sweet variety
Star apple fruit (*Chrysophillum albidum*)	1.14 mg/kg	[22]	very sweet variety
Pomegranate juices	0.13–0.37%	[37]	Juices were analysed from Wonderful and Mollar de Elche varieties of pomegranates. A third juice, referenced as “Coupage” was evaluated, consisting of a 1:1 mix of Wonderful and Mollar de Elche
Carrot	0.051 ppm	[38]	Concentration of p-cymene in headspace (0.25 L) collected from samples
Pine needle	0.003 ppm		
Tangerine	0.009 ppm		
Tangerine peel	0.408 ppm		
Strawberry	0.006 ppm		
Sepals of strawberry	0.003 ppm		
Orange juice	0.0003 ppm		
